# Editorial: Foundations of Theoretical Approaches in Systems Biology

**DOI:** 10.3389/fgene.2018.00290

**Published:** 2018-08-15

**Authors:** Alberto Marin-Sanguino, Julio Vera, Rui Alves

**Affiliations:** ^1^Specialty Division for Systems Biotechnology, Technische Universität München, Garching bei München, Germany; ^2^Laboratory of Systems Tumor Immunology, Department of Dermatology, Universitätsklinikum Erlangen and Friedrich-Alexander-Universät Erlangen-Nürnberg, Erlangen, Germany; ^3^Departament de Ciencies Mediques Basiques, University of Lleida, Lleida, Spain

**Keywords:** systems biology, network biology, mathematical modeling, computational modeling, systems medicine, biotechnology

The importance of systemic approaches in understanding biology was recognized as early as in the nineteenth century (Bernard and Dagonet, 2013). Around the 1920s and over the next few decades Briggs and Haldane (1925); von Bertalanffy (1962) and others Savageau (1969); Michaelis and Menten (2013) showed that such systemic views were both scientific and necessary in the biological sciences. Still, the only technology that could accurately perform biological studies integrating a large number of molecular components was mathematical modeling. This limitation remained in place until the late 1990s, making these studies hard to validate experimentally.

This pre-history of Systems Biology would end when full genome sequencing and the high throughput methods that would follow flooded every biological discipline with more data than could be analyzed. As a consequence, many discarded the usefulness of mathematical modeling under the assumption that there is no need to simulate what can be measured. Over time this view was understood as simplistic, and it became clear that mathematical and statistical modeling is essential to distill the sheer amount of molecular data available into “general biological laws” that explain how molecular components come together and form biological systems. We are leaving an era where large scale measurements of all molecular components in a cell dominated the field and entering a new wave of methodological development to integrate all those measurements into meaningful mathematical descriptions.

This integration needs to be multilevel. We need accurate methods that use experimental and qualitative information to perform whole-genome network reconstruction at the metabolic, signaling and the gene regulation level. We need general techniques that automatically derive and analyze mathematical models of such reconstructed networks. This Frontiers research topic, “Foundations of Theoretical Approaches in Systems Biology,” aims at paving the way to investigate if this set of approaches is mature enough to coalesce into a coherent body of knowledge.

In line with this, Torres and Santos introductory paper outlines the traditional modeling process as three-stage framework. In the first stage the biological system is framed as a conceptual model. In the second stage, the model is represented using a formal mathematical description. In the final stage the mathematical description is parameterized and studied through analytical and simulation methods to understand the dynamic behavior and regulation of the system. Lomnitz and Savageau recognize the limitations implicit to that classical approach. They describe a method in which all possible qualitatively different types of dynamical behavior, or phenotypes, of the system can be mapped from the conceptual representation and identify the parameter ranges that make each phenotype realizable. They also contribute a toolbox that enables modelers to try that method.

Other contributions to the topic describe and analyze the diversity of modeling being used and emphasize some of the commonalities and differences among them. At the level of network reconstruction, where little quantitative information is available, network centrality measures determined using graph theoretical approaches can help in identifying the key elements in the network, as is reviewed by Jalili et al.. As the causal structure of the network becomes clearer, logic modeling can offer testable dynamical and regulatory insights about the way in which, for example, signaling and gene regulation networks work (Khan et al., 2017). Abou-Jaoudé et al. review and discuss the potential of this type of modeling to reconstruct and analyze large, intricate biochemical networks.

Moving to models that describe biological systems using linear mathematics and steady state approximations, Müller and Regensburger explores the concept of elementary flux modes, a defining set for every possible flux distribution in a biochemical network. They use combinatorial mathematics and polyhedral geometry (Rockafellar, 1969) to propose alternative ways to search for flux modes in metabolic network analysis. Dolatshahi and Voit explore and discuss strategies for model parameters estimation that extend the use of *dynamic flux estimation* method for the analysis of metabolic time series data to general, slightly underdetermined metabolic networks. This method establishes a bridge between constraint-based models, which can be formulated with minimal information, and kinetic models that can be used to analyze transient data.

Hahl and Kremling examine the parallels and discrepancies between deterministic (ordinary differential equations) and stochastic approaches (chemical master equation) of molecular systems, discussing when to choose one or the other.

Overall, choosing a modeling framework is a trade-off that should consider the question being addressed as well as the data that is available to inform model creation. Models for bacterial lung infection (Cantone et al.) and cyanobacteria (Westermark and Steuer) are used to illustrate the advantages and disadvantages of alternative approaches, and to point out ways in which those approaches can be combined to create multi-level models.

Another important issue in mathematical modeling is that of model reduction. This is the process of identifying simpler but accurate enough versions of a larger model. Classical approaches to model reduction can be found in the field of enzyme kinetics. This field combines graph theoretical approaches with considerations about the differences between the characteristic time scale of individual chemical reactions or between the concentrations of the various species in a network to derive single equations that describe the dynamic behavior of fairly complex networks. Rosenblatt and coworkers (Rosenblatt et al.) present a graph-theoretical algorithm for deriving steady-state expressions by stepwise removal of cyclic dependencies between the network model variables. In parallel Löwe et al. and Koch et al. provide examples that illustrate the importance of choosing the appropriate mathematical formalism and how that formalism can be used to develop efficient approaches to model reduction.

Coming full circle, Kimura et al. illustrate that dynamic mathematical models can also be used for inferring network structure and refining the initial conceptual model on which the mathematical model is based.

Together, the collection of papers under the research topic “Foundations of Theoretical Approaches in Systems Biology” shows how theoreticians are exploring many different avenues to interpret experimental data and distill them into “biological laws.” In addition, this topic contributes to understand where those approaches overlap and where they complement one another. Only through such an effort can we avoid fragmentation and minimize duplication of efforts, and thus contribute to the consolidation of Systems Biology as a field of knowledge rather than an assortment of techniques.

## Author contributions

All authors listed have made a substantial, direct and intellectual contribution to the work, and approved it for publication.

### Conflict of interest statement

The authors declare that the research was conducted in the absence of any commercial or financial relationships that could be construed as a potential conflict of interest.
